# Multiscale Modeling of Light Absorption in Tissues: Limitations of Classical Homogenization Approach

**DOI:** 10.1371/journal.pone.0014350

**Published:** 2010-12-31

**Authors:** Stephane Mottin, Grigory Panasenko, S. Sivaji Ganesh

**Affiliations:** 1 CNRS, University of Lyon, University of Saint-Etienne, UMR5516, Saint-Etienne, France; 2 University of Lyon, University of Saint-Etienne, LAMUSE, Saint-Etienne, France; 3 IIT Bombay, Powai, Mumbai, India; University of Milano-Bicocca, Italy

## Abstract

In biophotonics, the light absorption in a tissue is usually modeled by the Helmholtz equation with two constant parameters, the scattering coefficient and the absorption coefficient. This classic approximation of “haemoglobin diluted everywhere” (constant absorption coefficient) corresponds to the classical homogenization approach. The paper discusses the limitations of this approach. The scattering coefficient is supposed to be constant (equal to one) while the absorption coefficient is equal to zero everywhere except for a periodic set of thin parallel strips simulating the blood vessels, where it is a large parameter 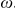 The problem contains two other parameters which are small: 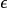, the ratio of the distance between the axes of vessels to the characteristic macroscopic size, and 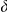, the ratio of the thickness of thin vessels and the period. We construct asymptotic expansion in two cases: 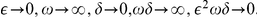 and 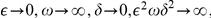 and prove that in the first case the classical homogenization (averaging) of the differential equation is true while in the second case it is wrong. This result may be applied in the biomedical optics, for instance, in the modeling of the skin and cosmetics.

## Introduction

### Physical background of the problem

In the present paper we consider the Helmholtz equation with rapidly oscillating large potential with a periodic support having a small measure of its intersection with a period. This absorption coefficient 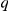 of the potential (more precisely, it is the ratio between the absorption coefficient and the diffusion coefficient) depends on three small parameters: 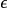 is a standard homogenization parameter that is the ratio of the period of the potential and the characteristic macroscopic size; 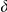 is the ratio between the measure of the intersection of the support of 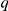 and the period; 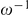 is a small parameter standing for the inverse of the ratio of the maximal value of the coefficient 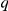 and the diffusion coefficient multiplied by the square of the characteristic macroscopic size of the problem (we will consider the case when 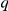 takes only two values: 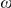 and 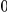).

The Helmholtz equation
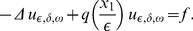
(1)is considered below as a model of the light absorption in tissues under hypothesis that this absorption takes place only in the set of parallel thin blood vessels (where 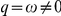) and this absorption is ignored outside of these vessels.

The linear dimensions of the vessels are much smaller than the linear dimensions of the body as a whole. Typically the distance between two neighboring large micro-vessels (of about 15 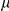
m in the diameter) is around 180 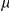
m in primate cerebral cortex [1]. This distance is also used in 3D simulation of tumor growth and angiogenesis [2]. Assume at the first approximation that the tissue is a nearly periodic structure. Moreover, consider the two-dimensional idealization of this periodic structure that is, the periodic set of the parallel strait narrow identic vessels separated by the homogeneous tissue. Let L be the macroscopic characteristic size and let 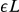 be the distance between two neighboring vessels (strips). It means that the parameter 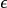 stands here for the ratio of the distance between two neighboring vessels and the characteristic macroscopic size 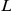. It is assumed throughout that this ratio is a small parameter. Indeed, if the characteristic macroscopic size (L) is equal to 10 mm (it is a typical value of the diffuse optical tomography [3]) and the distance between the vessels is equal to 0.18 mm, then 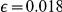. The second parameter of the model is the ratio of the thickness of the vessels and the distance between neighboring vessels denoted 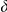. This 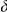 as well is supposed to be a small parameter. Thus, in the discussed above structure we have 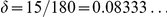. In the spectral window 500 nm–700 nm, the oxyhaemoglobin extinction coefficient shows a wide dynamic from 275 (mole/L)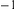 cm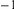 at 690 nm (the lowest) and 55 500 (mole/L)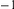 cm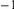 at 575 nm [4]. In order to quantify the maximum variation we compare arterial blood (90 percent saturated) with pure water at 690 nm and 575 nm then the ratio of the absorption coefficient is respectively 300 and 400000. At 700 nm the absorption of tissue (without blood) is about ten times less than that of the pure water [5]. In the visible window the maximum of this ratio can be more than 10000. That is why in the idealized model we consider the case when the absorption coefficient is equal to zero out of vessels. As we have mentioned above the light absorption process is described here by the Helmholtz equation (1), where 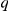 is just non-dimensionalized absorption coefficient, equal to zero out of vessels and equal to the great (dimensionless) parameter 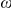 within the vessels. Let us remind that the physical sense of this great parameter is the ratio of the absorption and diffusion effects, more exactly, the ratio of the absorption coefficient and the diffusion coefficient multiplied (i.e. the ratio is multiplied) by the square of the characteristic macroscopic size 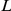. Let the haemoglobin concentration be 150 g/liter. Then in order to convert the molar extinction coefficient 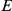 to the absorption coefficient (in mm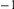), one has to multiply it by 0.00054. At the wavelength 575 nm for the scattering coefficient close to 2.5 mm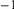, 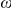 is about 23000. Then 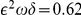 and 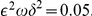 In the case of penetrating vessels with the average diameter close to 65 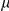
m, at the same wavelength, 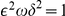. Then the case when 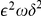 is greater than one can be found in a spectral window below 600 nm and for vessels of large diameter. In neurophotonics, the highly vascularized pie-matter correspond to the maximal values of 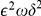. The highly vascularized tumors could be also a special case when 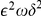 is very high.

Models of this “composite medium” are widely considered in biophysics. Let us give a short review of these results. The partial differential equation (PDE) diffusion with absorption is one of the most important in life science, and in particular, in tissue optics [6]. The researchers are interested in two types of results:

- when the optical properties are unknown, and they want to find them using observed measurements, it is the harder well-known inverse problem.
- when the optical properties are known, then they can use many strategies to calculate various quantities of interest (reflectance, transmission, fluence). This is often referred to as the forward problem.

But in all these approaches, the community of research in biophotonics uses the volumetric averaging of the absorption coefficient. This problem is crucial in the domain of in vivo diffuse optical tomography [3], [7], and is always present in *in vivo* optical neuromethods [8].

There are as well some experimental papers on the problem of averaging of blood absorption of light in tissue but without the homogenization theory [9]–[14].

Our goal in the present paper is to show that the **classical homogenization (averaging) approach** to the solution of equation (1) leading to the approximation
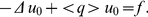
(2)where 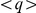 is often approximated to the volumic mean value of 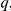 **has some limitations**. Indeed, we will show that it is right for some combination of magnitudes of parameters 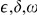: it can be proved that 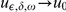 but it is inapplicable for some other combinations. In this paper we do not construct the expansions for all possible combinations; we only prove the classical homogenization result in the case
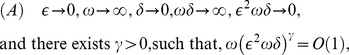
and we show that the homogeneous model (2) is inapplicable in the case
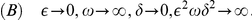



It means that the “diluting” of vessels in multiscale modeling of light absorption of tissues may be applied only in justified cases, in particular, in case A. In fact, the situations when the coefficients of equations depend on two or more small parameters and it leads to a fail of classical homogenization are not too surprising now: first results of this type could be found in [15]–[17]. For some cases the non-classical asymptotic expansions were constructed. However, to our knowledge, such examples have not been constructed for rapidly oscillating large potentials with narrow support. The present paper tries to contribute in this important case of biophysical applications.

In order to simplify technical details of the analysis we consider the boundary value problem for the Helmholtz equation set in a layer 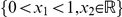 with the Dirichlet conditions on the boundary of the layer; we assume that the right-hand sides of the equation and of the boundary condition do not depend on 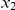. In this case we may seek a solution independent of 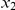 as well. The Helmholtz equation takes the following form:
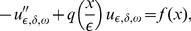
(3)where 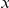 stands for 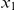, 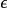 is a small positive parameter, such that, 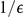 is an integer.

We consider a boundary condition corresponding to a constant solution in the case of the constant absorption coefficient 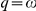 and of a constant right-hand side 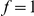. Then 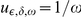. So, these boundary conditions are:
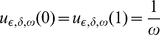
(4)


Similar problems were addressed by many authors [18]–[22]; however the potentials considered in these works are typically 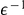 or 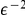. In this work, there are three parameters and we study all relevant asymptotic regimes. We follow the ideas and methods of [15]–[17], [23], [24] in the sense that we construct asymptotic expansions for 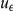 and analyze all the important asymptotic regimes of the parameters 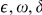.

Mention that the Helmholtz equation (1) (or (3)) is not a perfect model for the light absorption process: it is not more than an approximation of a more adequate model based on the radiation transfer equation [25]. Indeed, one can introduce the average (over all directions 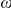) for the diffused radiation intensity 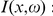


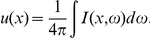



In the case of the described above plane geometry the diffusion approximation of the radiation transfer has a form [25]

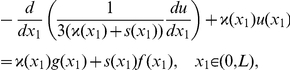
where 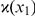 and 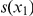 are the coefficients corresponding to the light absorption and dispersion respectively, 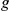 is the blood heat radiation intensity and 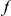 is the external radiation intensity, 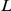 is the thickness of the irradiated skin strip, 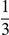 is the dimension factor. Introducing the new variable corresponding to the relative optical depth of the radiation process
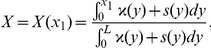
we get for 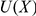 (here 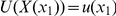):
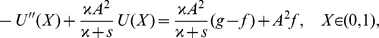
where 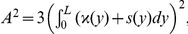 and functions 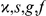 depend on 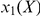, solution of equation
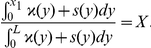



Making the change of unknown function
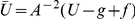
we get finally equation (3):
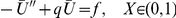
with 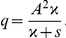


The choice of the boundary conditions (4) is not too important for our analysis: the asymptotic approach developed below can be applied in the case of other conditions, for instance,
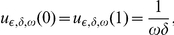
corresponding to a constant solution for equation (3) with 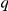 replaced by its volumic mean 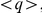 or
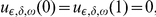
or the periodicity conditions (mention that the diffusion approximation of the radiation transfer may loose its precision near the boundary).

### Mathematical statement of the problem

Consider interval 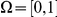 and let 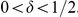 Let 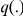 be a 1-periodic function defined on the basic period 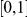 by
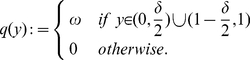
(5)


Let 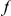 be smooth enough (this assertion will be formulated more precisely later). We are interested in studying the asymptotic behaviour as 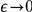, 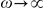, and 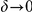 of 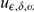 solving the following boundary value problem:
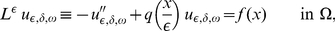
(6)

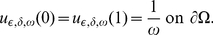
(7)Note that we have the following a priori estimate for solution of equation (6) with boundary condition (7) replaced by the homogeneous one
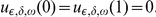

**Proposition 0.1.** *There exists a constant* 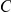 *independent of small parameters such that*

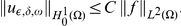
Here 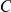 may be taken equal to 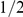. A well-known imbedding theorem gives:

**Corollary 0.1.** *There exists a constant* 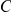 *independent of small parameters such that*

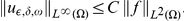
Applying equation, we get:

**Corollary 0.2.** *There exists a constant* 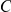 *independent of small parameters such that*

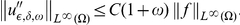



Returning now to equation (6) with non-homogeneous boundary condition (7) we can present the solution as a sum of the solution of equation (6) with homogeneous boundary condition and the solution of the homogeneous equation (6) with non-homogeneous boundary condition (7). Applying the maximum principle argument, we get:

**Proposition 0.2.** *There exists a constant* 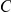 *independent of small parameters such that solution of problem (6)-(7) satisfies:*

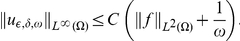

**Remark 0.1.** If boundary condition (7) is replaced by another one:
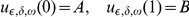
with some numbers 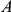 and 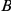 then the estimate of Proposition 0.2 takes form:
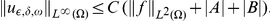



Let us mention one more useful inequality for functions 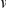 of 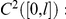


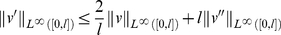
(8)


Proof: For any 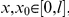 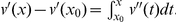 Choose 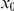 such that 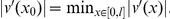 We get:
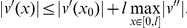



If 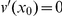, then the estimate is proved. If 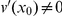, then the sign of 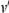 is constant on the whole interval 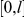; consider the case 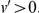 Then 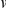 is increasing and 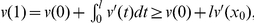 and so,
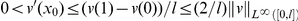
and we get (8). In the same way we consider the opposite case when 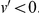


## Methods

### Asymptotic expansion in the case 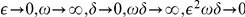


Consider the 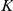-th level approximation 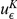 of 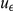 given by an ansatz from [15]:
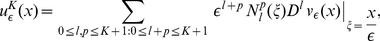
(9)where 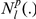 are 1-periodic functions, 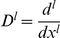, and 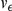 is a smooth function which will be sought in a form
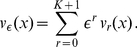
(10)Substituting the expression for 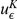 from (9) in (6) we obtain
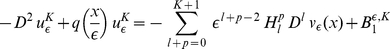
(11)where
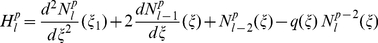
(12)and 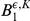 is given by
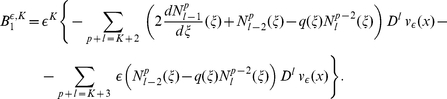
(13)In writing expressions (11), (12) we followed the convention that 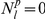 if at least one of the two indices 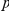 or 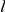, is negative.

We choose 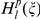 being a constant such that the equation (12) has a solution, i.e.
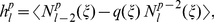
(14)where 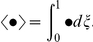 Also, periodic solutions 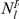 of equation
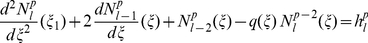
(15)are chosen to be of average zero for all 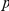 and 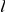 such that, 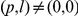; 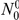 is chosen equal to 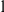. We get: 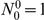, 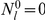, 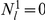 for all 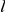, 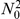 is a 1-periodic solution of equation
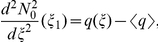


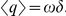



We solve the family of equations (15) by determining for each fixed 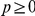 the solutions 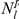 for all 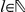. Due to the form of the equation (15) the behavior of 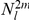 and 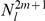 will be similar.

With this background, we now state the principal result concerning the 1-periodic functions 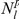.

**Lemma 0.1.** Let 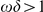. *For each* 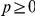 *(of the form* 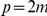 *or* 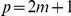 *) and* 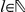,

1. *Functions* 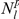 *are piecewise polynomials on* 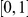 *with respect to the partition* 
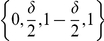
.
2. *There exists a constant* 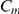 *independent of the small parameters such that, for* 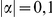, 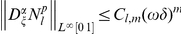, 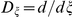, *and* 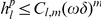.

**Proof.** The proof is by induction on 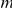.

**Step (i).** Let 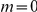. That is, 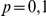. We get: 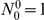, 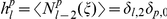, 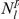 satisfy equations (15) with the last two terms equal to zero; so 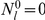, for all 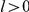, 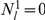 for all 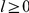 and the assertion of lemma for 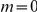 is evident.

**Step (ii).** Let 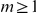 and let all 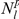, 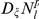 and 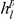 with 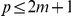 be bounded by 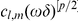, where 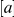 stands for the integer part of 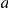, and 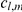 is a constant independent of the small parameters. Consider equation (15) for 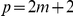. For 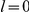 it takes form
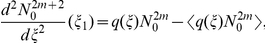
with the piecewise polynomial right hand side; moreover, the primitive (with vanishing mean value) of this right hand side is also bounded by the same order. That is why, integrating this equation twice and keeping every time the vanishing mean for the primitive, we prove the assertion of lemma for 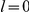. In the same way we prove it for 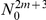.

Now we apply again (15), express 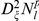 from this equation and prove by induction on 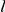 that all 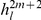, 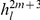, the right hand sides and so, finally, 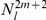 and 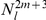 are bounded by 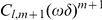. The lemma is proved.

Substituting the expression for 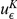 from (9) in (7) we obtain
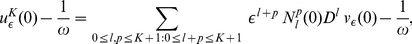
(16)

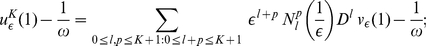
and applying the 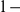
periodicity of functions 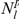 and the divisibility of 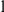 by 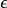 we get:
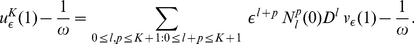
(17)


Substituting the expression for 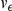 (10) in (11),(16),(17), we get:
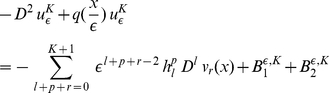
(18)and
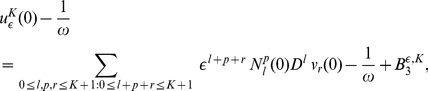
(19)

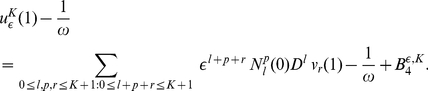
(20)


Here
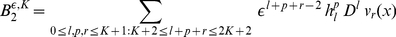
(21)

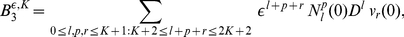
(22)and
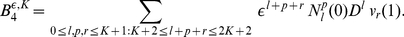
(23)


Define now functions 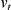 from boundary value problems:
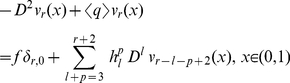
(24)and
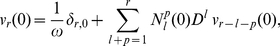
(25)

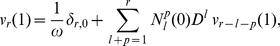
(26)Here 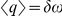 is supposed to be greater than 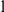. These equations have a form
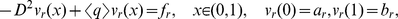
where 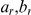 are some real numbers and 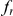 is a smooth function. In the same way as in the first section we prove that
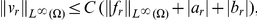


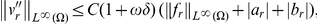
and so,
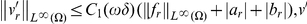
where the constants 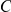 and 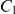 are independent of small parameters.

Applying the induction on 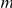, we get:
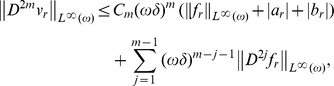
where the constants 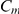 are independent of small parameters. Applying now (8), we get: for 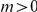


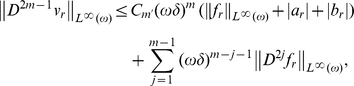
and so,
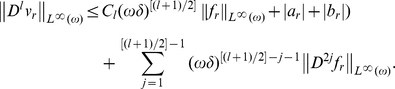



**Remark 0.2.** Functions 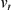 depend on small parameters but their asymptotic expansion can be constructed by classical boundary layer technique (see [23]). For instance, if 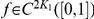, then 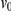 has a form:
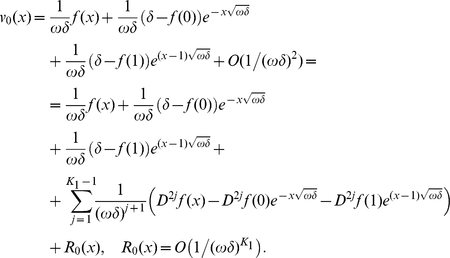



Moreover, if 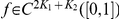, then 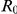 is 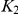 times differentiable and satisfies the estimate (see the above a priori estimate for 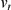):
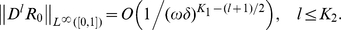



In the same way we construct by induction the expansions of functions 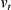, 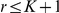. Assume that 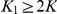, 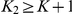. Consider the right hand side 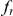 having the form of a linear combination with some bounded coefficients 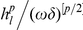 of functions 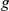 having a form:
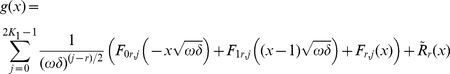
where 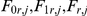 are 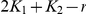 times differentiable functions independent of small parameters 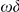 and 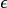, such that, there exist constants 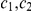 independent of small parameters satisfying for any real 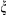 inequalities
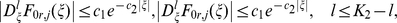




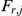
 are 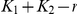 times differentiable on 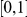,
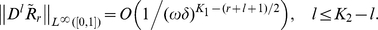



The right hand sides of the boundary conditions as well have a similar form: they are some linear combinations with bounded coefficients 
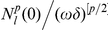
 of constants 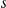 and 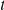 having a form:
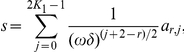


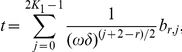
where 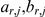 are independent of small parameters.

The expansion of 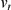 has a similar form of a linear combination with bounded coefficients of functions
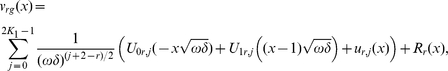
where 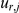 satisfy equations (functions with the negative indices are equal to zero)
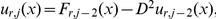
and exponentially decaying at infinity functions 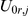 satisfy equations
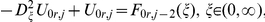
and boundary conditions
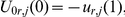
exponentially decaying at infinity functions 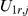 satisfy equations
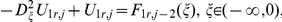
and boundary conditions
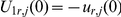
and of the exponents
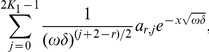


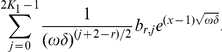



Here
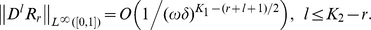



Applying the induction on 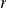, the explicit expressions for the right hand sides of the problems (24),(25),(26) for 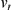 and the estimates of Lemma 0.1 we prove that there exist constants 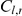 independent of small parameters, such that,
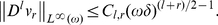
(27)


This estimate and Lemma 0.1 are crucial for evaluation of the discrepancies 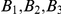 and 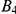:
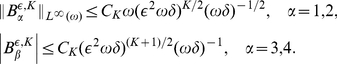
(28)


Now, applying the a priori estimate of the Remark after Proposition 0.2 we get the estimate
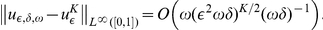



Assume that there exists a positive real 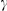 such that, 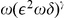 is bounded by a constant independent of small parameters. Then the last bound yields:
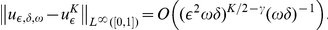



Taking 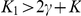, we get:
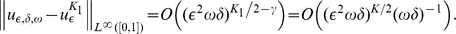



On the other hand, (27) and Lemma 0.1 give:
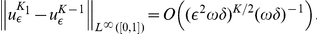



So, from the triangle inequality we get
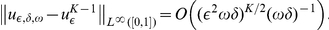

**Theorem 0.1.** *Assume that there exists a positive real* 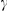 *such that,* 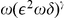 *is bounded by a constant independent of small parameters. Let* 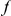 *belong to* 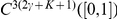. *Then there exists a constant* 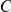 *independent of small parameters such that*

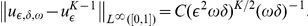



Consider now the very first term of the expansion 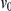 and 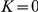 we get:

**Corollary 0.3.** *There exists a constant* 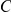 *independent of small parameters such that*

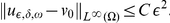



Mention that the error of order 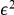 is much smaller than 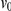 that is of order 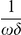, and so the relative error of the approximation of the exact solution 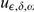 by 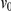 is small.

**Remark 0.3.** Here we have considered the case when 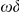 stands for a large parameter tending to the infinity. The case when 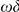 is a positive finite constant may be considered by a classical technique [15] and the estimate of Corollary 0.3 becomes
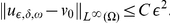



### Asymptotic analysis: case 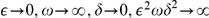


Here we will construct an example where the behavior of the solution is completely different from the behavior described in the previous section. In particular, the solution of the problem is different from the behavior of the solution 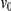 of the homogenized equation. For the sake of simplicity consider the right hand side 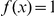. Consider an auxiliary function 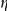 defined on the interval 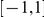 as a function from 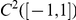 independent of small parameters such that it equals to zero on the interval 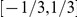 and it equals to one on 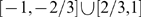. Let us keep the same notation 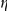 for the 2-periodic extension of this function on 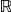. Consider an approximation for 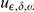 having a form:
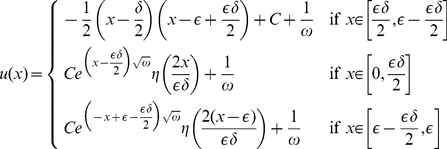
(29)where 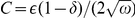 and extend it 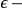
periodically to 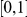. By a simple calculation we find that 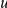 satisfies the boundary conditions exactly and the equation with a discrepancy of order 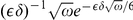, i.e. 
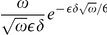
. Assume that there exists a positive real 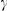 such that, 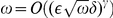. Then for any positive 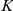, 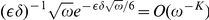.

So, the discrepancy of the equation is 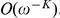 Applying now Corollary 1.1, we get an error estimate in 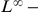
norm of the same order:
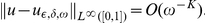



Mention that for 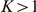 the right hand side of this bound is much smaller than the values of approximation 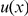, and so the relative error of the approximation of the exact solution 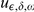 by 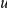 is small.

## Results and Discussion

### The main observations on the asymptotic analysis

We record some observations in the following remark.

**Remark 0.4.** Let us compare the asymptotic behavior of solution for 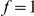 in the cases
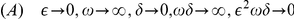
and
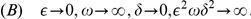



In the case (A), the leading term is equal to the solution 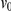 of the homogenized equation (2), that is the constant 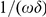 plus two exponents rapidly decaying from the boundary: their contribution can be neglected at the distance of order of 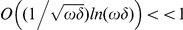. In the case (B) the approximate solution is completely different: it is 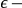
periodic piecewise quadratic function of order 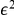 except for the small intervals where the potential is large. That is why the homogeneous model (2) is inapplicable in this case.

Thus, the homogenized model (2) may be applied only in the case 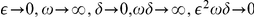, when it is justified theoretically. The homogenized model may be inapplicable in the case if 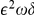 is not small.

### On the effective absorption coefficient, the volumetric mean and the total absorption coefficient

Biological tissues are highly heterogeneous media at the microscopic scale. For years, the patterns of the mammalian cortex micro-angioarchitecture have attracted the interest of many research groups [1], [26]–[28]. At different scales the blood vessels have very different physical characteristics. Moreover the absorption coefficient is great in the vessels and small out of the vessels in UV and visible parts of the spectrum. At the end they induce multiscale complex photon propagation and absorption. On the other hand the experimental data are obtained mainly at the macroscopic scale, and so one of the main questions is how to make a passage from the micro-scale to the macro-scale. The main mathematical tool of such passage from one scale to another (the up-scaling) is the homogenization theory, see [15], [19], [23], [24] and the references there. Normally, the microscopic description of the heterogeneous medium can be replaced by an equation with constant coefficients. This equation is called the homogenized equation and the constant coefficients are effective coefficients. This homogeneous approximation for the heterogeneous medium is justified if the solutions of both models are close in some norms. In some cases a heterogeneous medium cannot be approximated by a homogeneous one [16], [17], [23], [24]. Then the notion of an effective coefficient cannot be introduced in the above sense.

In particular, in the present paper we prove that if product 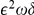 is small then the homogenized model (2) is justified and the effective absorption coefficient is equal to **the volumetric mean value** 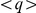 of function 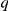. The smallness of the value of this product 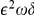 means that the relative error of the approximation of the heterogeneous medium by the homogeneous one is of order of this product. The effective absorption coefficient is an important quantity because the detailed knowledge of the tissue macroscopic optical properties is essential for an optimization of optical methods i.e. for modeling the color of skin, of port-wine stains and for tumor detection; it helps to adapt an appropriate photodynamic therapy, in particular, some laser treatment.

On the other hand the present paper shows the limitations of the homogenized models for the absorption problems. Indeed, if product 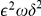 is not small then the homogenized model (2) as well as other homogenized models (with other possible constant values of the effective absorption coefficient) do not approximate the initial microscale model, because the solution of problem (2) does not oscillate for any choice of the effective absorption coefficient, while the solution of equation (6) with boundary condition (7) rapidly oscillates (see (29)).

This theoretical argument is confirmed by some physical reasons and by experimental observations. Many authors [10], [12], [14], [29] show that the assumption of a homogeneous distribution of blood in the tissue may strongly overestimate the total blood absorption when absorption is high and/or the vessels have sufficient diameter. For large vessels less of light reaches the center of the vessel, and the absorbers in the center of a vessel contribute less and less to the total attenuation of the light. However, the total blood absorption is an important qualitative characteristic of the absorption process and so it should be calculated with a great precision. Let us apply the above asymptotic analysis provided for equation (6) with boundary condition (7) and calculate the total blood absorption in cases A and B. Let us define the total absorption coefficient 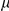 as a ratio of the integrals
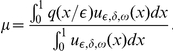



For the particular case of a constant coefficient 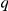 we get evidently 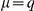. In the case A substituting the leading term of an asymptotic expansion we get that the asymptotic behavior of 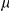 is given by an approximate formula 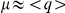, i.e. it is close to the volumetric mean of 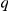 that is, 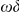. In the case B the value of this total absorption coefficient 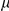 is much less than the volumetric mean (that was observed in the discussed above experiments): the direct computations for the approximation (29) show that 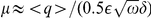 with 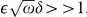


The authors of papers [10], [12], [14], [29] characterize the fall of the total absorption coefficient by the correction factor 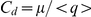 and discuss the situations when this factor is different from 1. In our asymptotic analysis we see that in the case A 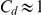 but in the case B 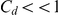.

We hope that our result will help to analyze the link between the total absorption coefficient 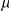 and other parameters which may be applied in optical tomography [3], [7].
